# Fully automated high-throughput immuno-µPlaque assay for live-attenuated tetravalent dengue vaccine development

**DOI:** 10.3389/fimmu.2024.1356600

**Published:** 2024-02-12

**Authors:** Yi Wang, Matthew C. Troutman, Carl Hofmann, Ariel Gonzalez, Liping Song, Robert Levin, Heidi Yoder Pixley, Kristine Kearns, Pete DePhillips, John W. Loughney

**Affiliations:** ^1^ Analytical Research & Development, Merck & Co., Inc., Rahway, NJ, United States; ^2^ Biostatistics, Merck & Co., Inc., Rahway, NJ, United States; ^3^ Vaccine Drug Product Development, Merck & Co., Inc, Rahway, NJ, United States

**Keywords:** dengue, live virus vaccines, multivalent vaccines, potency test, immuno-µPlaque assay, high-throughput analytics, integrated robotic system, lab automation

## Abstract

Dengue fever has remained a continuing global medical threat that impacts half of the world’s population. Developing a highly effective dengue vaccine, with live-attenuated tetravalent vaccines as leading candidates, remains essential in preventing this disease. For the development of live virus vaccines (LVVs), potency measurements play a vital role in quantifying the active components of vaccine drug substance as well as drug product during various stages of research, development, and post-licensure evaluations. Traditional plaque-based assays are one of the most common potency test methods, but they generally take up to weeks to complete. Less labor and time-intensive potency assays are thus called for to aid in the acceleration of vaccine development, especially for multivalent LVVs. Here, we introduce a fully automated, 96-well format µPlaque assay that has been optimized as a high-throughput tool to evaluate process and formulation development of a live-attenuated tetravalent dengue vaccine. To the best of our knowledge, this is the first report of a miniaturized viral plaque method for dengue with full automation via an integrated robotic system. Compared to the traditional manual plaque assay, this newly developed method substantially reduces testing time by approximately half and allows for the evaluation of over ten times more samples per run. The fully automated workflow, from cell culture to plaque counting, significantly minimizes analyst hands-on time and improves assay repeatability. The study presents a pioneering solution for the rapid measurement of LVV viral titers, offering promising prospects for advancing vaccine development through high-throughput analytics.

## 1 Introduction

Dengue is a systemic viral infection transmitted from mosquitoes to humans. The rapid spread of dengue virus has become an increasing global medical burden across approximately 100 countries, causing 100-400 million dengue infections each year and threatening half of the world’s population according to the World Health Organization (WHO) (1–4). Currently, there is no specific treatment for dengue. Thus, developing a safe and efficacious dengue vaccine remains one of the essential solutions for this global health issue (5–7). Merck & Co., Inc. Rahway, NJ, USA; formulated a live-attenuated tetravalent investigational dengue virus vaccine from TV003/TV005 developed by the National Institute of Allergy and Infectious Diseases (NIAID), which has received promising clinical study results (8–10). During the development of a live virus vaccine (LVV), potency is one of the critical quality attributes needed for release testing to measure the immunogenicity of vaccine samples and to ensure the effectiveness, safety, and consistency of vaccine products (11–13). Potency methods mainly fall into three categories: infectivity, relative potency, and antigen mass methods. Infectivity is the most common method and can be further categorized into plaque assay, tissue culture infectious dose 50 (TCID_50_), cell culture infectious dose 50 (CCID_50_), animal Lethal Dose 50 (LD_50_), and focus-forming assays (FFA), etc (14). Each infectivity assay has its own advantages and disadvantages regarding aspects such as time, cost, throughput, and ease of method transfer, which all need to be taken into consideration when selecting methods for potency measurements.

Plaque assays utilize the formation of discrete visible structures (i.e. plaques) formed throughout cell monolayers upon viral inoculation. These methods have long been used to directly quantify virus titers and viral neutralizing titers of human serum (15–17). A plaque assay normally consists of cell plating, viral absorption, subsequent viral replication, and cell-to-cell spread, followed by detection and counting of plaques. In traditional assays, plaques need to grow to sizes that can be easily seen by eye for the purposes of counting. Due to the size requirement for visual counting, formats for these methods range from single petri dishes to 24-well plates (18, 19). For similar reasons, a plaque assay varies, depending on the virus used, from a time scale of days for fast-replicating viruses to weeks for slowly-replicating viruses, which inherently creates bottlenecks in analytical workflows during LVV development. FFA, on the other hand, detects infected cells, or cell clusters, and the associated infectious viral particles (i.e., foci) before an actual plaque is formed. In FFA, focus-forming units (FFU) are typically smaller in size as compared to viral plaques, necessitating the use of alternative staining techniques such as immunostaining to label viral antigens and quantify them discretely. This approach offers notable advantages including the use of a smaller assay plate format and a shorter assay duration, rendering it well-suited for high throughput applications and highly desirable for expeditious product development (20, 21). Recent developments in these analytical methods include evaluating novel overlay systems (22), improving the assay throughput, and advancing plaque staining, imaging, and calculation methods (23–25). Wen et al. published a higher throughput immunofluorescent imaging-based plaque method for measuring infectious respiratory syncytial virus (RSV), utilizing 96-well microplate formats to increase the number of samples tested per run. A plate washer was applied and proven consistent with manual washing to limit analyst-engaged operation time. Assay throughput was further improved by clear plaque visualization from fluorescent immunostaining and an automated plaque counting algorithm. The miniaturization of the assay to a 96-well format and sensitive immunostaining allowed the infection time to be reduced from 5 to 3 days (26). Another application of the high-throughput immuno-plaque assay was on SARS-CoV-2, reported by Amarilla et al. in 2021 (27). This work included an assay optimization study for viral titer measurement utilizing 96-well microplates and demonstrated a relatively less sensitive but suitable 384-well microplate format method for inhibition tests.

These studies have shown that laboratory automation devices, such as plate washers and multi-modal imagers, can accelerate potency testing, which can be broadly applied to support the development of biotherapeutic and vaccine development processes. Nonetheless, the complex experimental requirements of developing multivalent LVVs such as a tetravalent dengue vaccine remain numerous and daunting, which calls for further advancement in developing high-throughput potency methods. Particularly, optimization of viral replication within vaccine upstream cell culture vessels can create large design space. Fermentation steps take multiple days in many cases, and identifying an optimum time point is critical to streamline vaccine production (28). Design of experiments (DoE) studies for optimizations of parameters such as starting multiplicity of infection (MOI), incubation temperature, and pH also generate a large number of samples for potency measurement. Multivalency of the LVVs, such as dengue, further increases sample amount by folds. Likewise, the optimization of downstream processing through purification of viral particles can consist of multiple steps that need to be optimized for removal of impurities and to maintain sufficient infectious yield. Moreover, the optimization of a lyophilized multivalent LVV drug product is often accomplished by measuring single conditions over the additional factor of *time* to show stability (29).

Fully automated high-throughput assays offer accelerated analytical testing and ultimately expedited product development. There are also additional benefits, such as reduction in labor and long-term costs, improvement in reproducibility and safety when compared to manual methods (30, 31). Integrated robotic systems offer viable solutions towards enabling full automation across multiple complex processes, such as cell culture, liquid handling and, ultimately, fixation, immunostaining, and plate imaging. These systems have been documented to expedite diverse projects within the pharmaceutical industry (32, 33). Nevertheless, to the best of our understanding, there is currently no published study that has applied the full-automation approach to potency measurement of LVVs. Due to the multiplicative nature of the analytical demand for LVV products such as a dengue vaccine, we have developed a 96-well microplate-based fully automated high-throughput FFA to measure the absolute titer of dengue samples across the bioprocess design space. Viral protein immunostaining was carried out automatically and imaged to visualize the subsequent foci resulting from each serotype’s infection of Vero cells. We branded this FFA method “µPlaque” as a quicker, orthogonal method, as compared to the traditional 24-well plaque assay that requires manual staining and counting. This miniaturized, higher throughput “µPlaque” assay provides a much-needed acceleration to vaccine development cycles that require specificity, sensitivity, and functional assessment of LVV samples.

## 2 Materials and methods

### 2.1 96-well µPlaque assay

**Figure 1:**
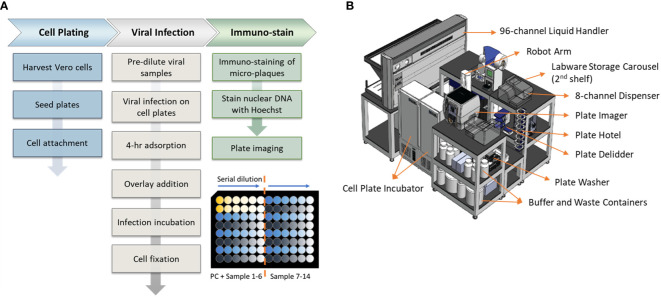
Assay workflow and automation setup. **(A)** Automated high-throughput µPlaque assay procedure. Bottom right plate layout shows serial dilution on a 96-well microplate. Each plate contains two positive controls (PC) and 14 vaccine samples with 6 serial dilutions of each sample. **(B)** A 3.3m (Length) × 2.4m (Width) × 2.3m (Height) automation system that µPlaque assay was developed on. Instruments including a liquid handler, incubators, dispensers, and readers were utilized to perform the assay from viral infection to cell fixation and immunostaining.


An overview of the assay workflow from cell plating to viral infection and immunostaining is described in **Figure 1A**. Vero cells are obtained from ATCC (#CCL-81, VA, USA). Cells are maintained on a CompacT SelecT (Sartorius AG, Germany) using T-175 flasks (Corning Inc., NY, USA) and grown in Gibco OptiPRO SFM (Thermo Fisher Scientific, USA) supplemented with 2% L-glutamine (Corning). On the day before viral infection, Vero cells are first harvested and seeded in 96-well Corning Tissue Culture (TC)-treated clear-bottom black microplates at 40,000 cells per well at 37°C, 5% pCO_2_, >80% rH. After overnight incubation to allow cellular attachment, cell supernatant is removed by gentle aspiration, and 25 µL/well diluted DENV samples are added to all wells. Viral adsorption proceeds for 4 hours in the incubator at the aforementioned incubation conditions. Following viral adsorption, 175 µL/well of pre-warmed overlay medium (OptiMEM + GlutaMAX, 1% Methylcellulose, 2% FBS, 20 µg/mL Ciprofloxacin, 2.5 µg/mL Amphotericin-B) is added to all wells without removal of inoculum. Viral spread occurs for 2 to 3 days, depending on the serotype, and then the overlay medium is removed by aspiration. Cells are fixed for 30 minutes with 3.7% formaldehyde in PBS. Cells are subsequently permeabilized with 0.5% Triton X-100 in PBS for 20 minutes, stained with Hoechst 33342 (Life Technologies, CA, USA), and blocked with 1% BSA in PBS for 30 minutes. Immunostaining is accomplished using type-specific rabbit monoclonal antibodies (developed internally and proprietary to Merck & Co., Inc., Rahway, NJ, USA) diluted to 1 - 2 µg/mL in Staining Buffer (PBS, 1% BSA, 0.05% Tween-20). Primary mAbs bind for 60 minutes and are detected using donkey anti-rabbit Alexa Fluor 488 (Jackson Immunoresearch, PA, USA). After immunostaining, viral plaques are imaged using a PerkinElmer (MA, USA) EnSight and plaques are counted using PerkinElmer Kaleido software. Viral titer or Potency of each sample was calculated using the following equation:

$$Viral\;titer\;\left(\frac{PFU}{mL}\right)=\frac{Counted\;Plaque\;Number}{Inocolum\;Volume\;\left(mL\right)}\times Total\;Dilution\;Factor$$

### 2.2 Automation of the µPlaque assay

The µPlaque assay was fully automated via a CompacT (Sartorius) cell culture system and an integrated robotic system by HighRes Biosolutions (HRB, MA, USA). The latter integrated system consists of multiple devices enclosed within a Class II, Type B2 enclosure (**Figure 1B**). The system includes a live cell plate incubator from LiCONiC (Liechtenstein), and an Ambistore (HRB) used for post-fixation plate incubations and labware storage (e.g., microplates and pipette tips). A Tecan (Switzerland) EVO liquid handler with a 96-channel pipette head was used for serial dilutions of viral samples within viral plates, viral addition from viral plates to cell plates, overlay addition, cell fixation, and immunostaining. A BioTek MultiFlo dispenser from Agilent (CA, USA) was utilized for cell permeabilization, Hoechst-block, and PBS addition steps. BioTek 405 LS plate washers were used for all plate washes in between staining reagents. An 8-slot plate hotel was included for short-period plate incubations. HRB PlateOrient and LidValet were also integrated in the system to optimize throughput by reorienting and lidding/delidding plates as they were processed. An ACell robotic arm (HRB) was installed on a linear rail within the integrated system to transfer labware among the instruments described above.

### 2.3 24-well manual plaque assay

The 24-well manual plaque assay is an immuno-plaque method in which vaccine samples and positive controls are pre-diluted based on the sample’s estimated titer before being subjected to a serial dilution and inoculated onto Vero cells that were seeded into 24-well plates prior. Following the inoculation step, samples and positive controls are allowed to incubate for 1 hour at 37°C, at which point the cells are overlayed with growth medium supplemented with methylcellulose and incubated for an additional 6 days. After the incubation, viral plaques are visualized upon cell treatment with serotype-specific anti-dengue monoclonal antibodies (proprietary to Merck & Co., Inc., Rahway, NJ, USA) followed by a horseradish peroxidase (HRP) conjugated secondary antibody (SeraCare Life Sciences, MD, USA). A 3,3’-Diaminobenzidine (DAB) substrate (Vector Laboratories, CA, USA) is then used to illustrate the plaques. Titers of test articles and positive control are determined by plaque counts in wells. This assay was developed following WHO guidelines for plaque-reduction neutralization testing of dengue virus antibodies (34).

### 2.4 Statistical analysis

Unpaired t-tests with Welch correction were employed to evaluate the statistical significance between two groups of data for µPlaque image analysis and tetravalent investigation. The analysis was conducted using GraphPad Prism 9.4.1 software. A 95% confidence interval was used in the tests, and the assumption of equal population variances was not required. The t-test was first conducted to compare manual and automated counting methods for µPlaque images. Subsequently, the statistical test was applied in the tetravalent study to assess monovalent versus tetravalent samples in both averaged plaque sizes and viral titers. 

A concordance study was performed between µPlaque assay and manual plaque assay. Thirty-six tetravalent formulation samples were tested in both the 24-well plaque method and the 96-well µPlaque assay to assess the correlation between the two methods across a broad range of virus concentrations (estimated 2E3-4E4 PFU/mL). Each sample was tested four times for individual serotypes with 2 replicates included in each test. This set of samples covers three different formulations with each formulation prepared at 6 different drug product concentrations in both liquid and lyophilized formats. Deming regression was performed for each DENV serotype assuming both x (plaque) and y (µPlaque) have measurement errors. Different uncertainties were assumed for the plaque and µPlaque assays using standard deviations of each assay as the x or y errors. All titer results were log_10_ transformed and utilized in the regression. The 95% confidence interval (CI) for the slope of regression for each serotype was calculated to determine the concordance between the two datasets.

## 3 Results and discussion

End-to-end automation of a high-throughput µPlaque assay was achieved via an integrated robotic system in a 96-well microplate format. The assay was further optimized for the development of a tetravalent dengue vaccine. Immunostaining was accomplished via type-specific anti-E protein antibodies, and detected features (referred to as *plaques* throughout this manuscript instead of *foci* to make a direct comparison with the 24-well plaque method) were subsequently counted and utilized to report the viral titer (35). Sample throughput was significantly improved from 32 samples per run in the standard potency method, a 24-well manual plaque assay, to 384 samples per run in the µPlaque assay with 6 dilution points for each sample in both assays as compared in **Table 1**. Assay duration was shortened by 3 to 4 days which further improved the assay throughput. µPlaque assay results and analysis, optimization, and analytical characterization parameters such as precision and repeatability are discussed below.

**Table 1:** Assay throughput comparison.

Platform Parameter Comparison	Manual (24-well)	µPlaque (96-well)
Samples/plate	4	16
Plates/run	8	24
Samples/run	32	384
Full Time Employee for Infection, Fixation, Staining	2	1
Plaque Counting Method	By eye	Automated plate imager & Counting algorithm
Assay duration	8 days	4-5 days depending on serotype

### 3.1 Imaging results

**Figure 2:**
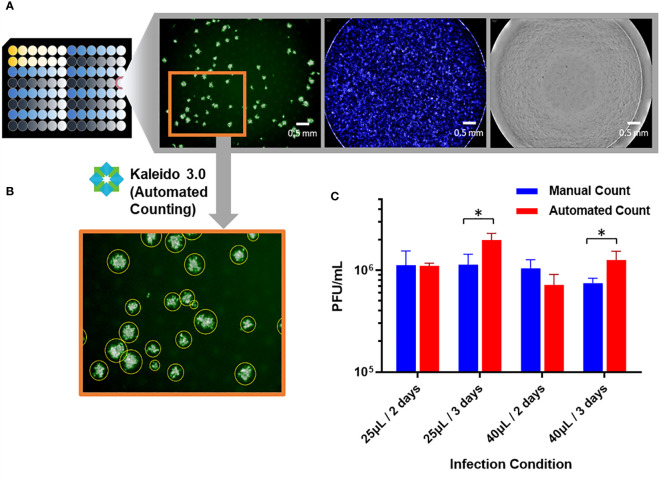
µPlaque imaging results and analysis via an automated counting algorithm. **(A)** Example images from one well of the 96-well microplate. Three images from left to right: GFP, DAPI, and brightfield. Scale bars are 0.5 mm for all three images. **(B)** Example of automated counting: Identified plaques are indicated by the yellow circles drawn around the particles. **(C)** Accuracy of automated counting. Two samples were tested under each condition with three dilution points of each sample. Four experimental conditions including 25 µL and 40 µL inoculum volumes, 2 days and 3 days viral progression times were tested with DENV-2 samples. Statistical analysis was performed between manual and automated counting for each of the infection conditions. Significant difference with 95% confidence level (P ≤ 0.05) was noted with *.

Viral titer of samples was reported as plaque-forming unit (PFU)/mL which is dependent on total plaques counted in each well image. **Figure 2A** shows an example of the results from one plate well imaged under excitation wavelengths of 385nm for Hoechst dye, 465nm for Alexa Fluor 488 dye, and brightfield using a multimodal plate reader. Both the brightfield and the Hoechst stain images confirm a valid confluent cell monolayer throughout viral infection and plaque growth processes. Alexa Fluor 488 dye-stained plaques exhibited distinguishing patterns under the green fluorescent protein (GFP) channel. Automated plaque counting was achieved utilizing Kaleido 3.0 method *Analysis of Nonlytic Virus Plaques* as shown in **Figure 2B**. Specific size thresholds were customized in the counting protocol to eliminate small patterns (e.g. debris, fibers, water droplets, etc.) that are unlikely to be plaques and to account for fused plaque particles. Accuracy of the counting algorithm was evaluated with a dataset of DENV-2 samples under four different experimental conditions including 25 µL and 40 µL inoculum volumes, 2-day and 3-day viral progression times. 4-hr adsorption time and 40,000 cell-per-well seeding density were applied to all conditions for consistency. Comparison between manual counting and automated counting results in **Figure 2C** indicates that the automated counting algorithm can either overcount or undercount the number of plaques depending on experimental conditions. T-tests were performed for all four conditions with P value results of 0.944, 0.018, 0.133 and 0.028 accordingly. Significant difference with 95% confidence level (P ≤ 0.05) was noted with * in **Figure 2C**. 25µL/2 days condition appears to be optimal with the least variance between manual and automated counting, thus was selected as the assay parameter for DENV-2. These parameters including inoculum volume and incubation time affect plaque counting by impacting the plaque size and density in individual wells, which further impacts automated counting results and the accuracy of data reporting (36). Thus, we carried out further assay optimization for all four DENV serotypes as follows.

### 3.2 Assay optimization

**Figure 3:**
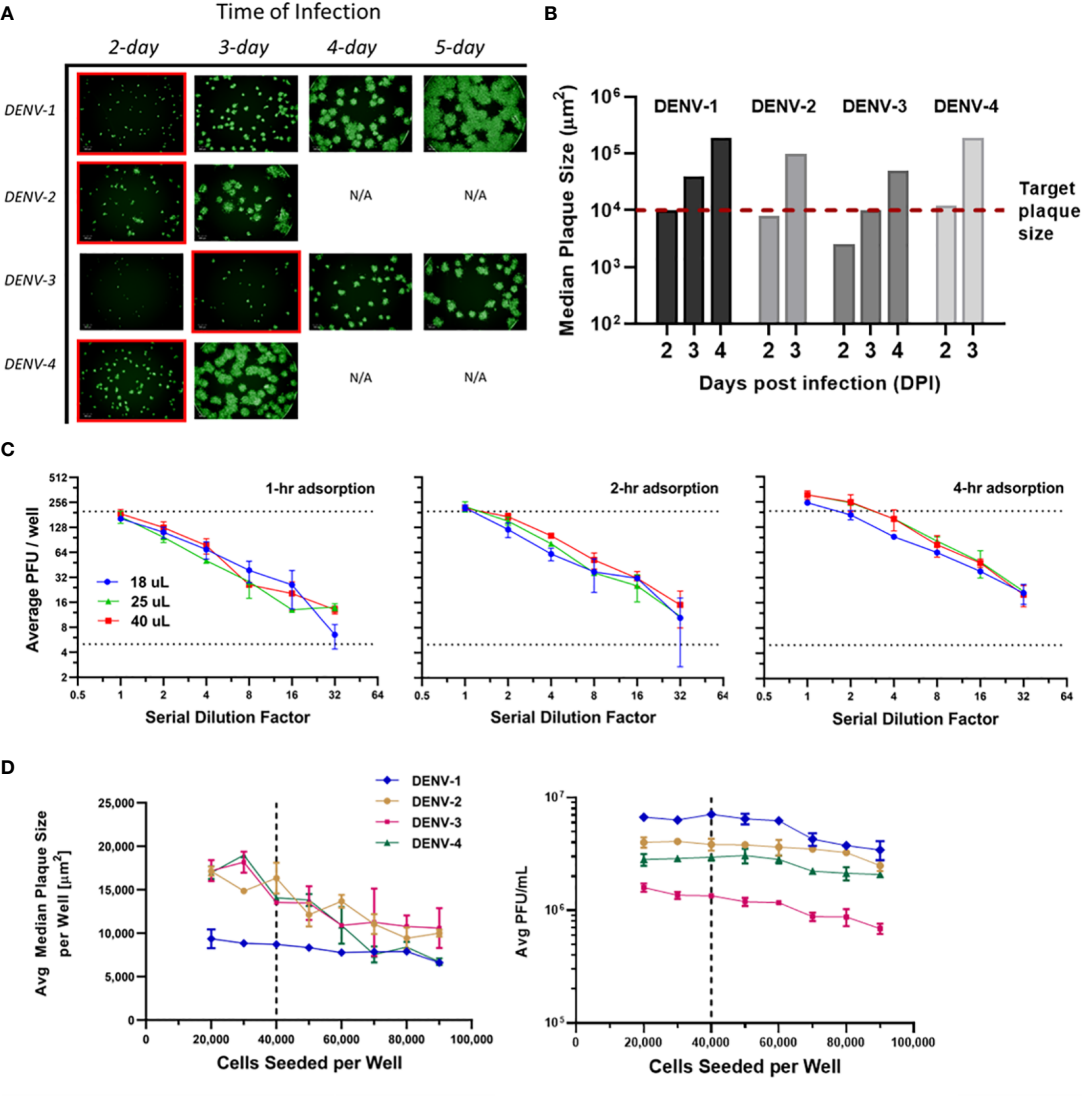
µPlaque assay optimization. **(A)** Progression of viral spread in representative wells from 96-well microplates. Images at selected time of infection based on median plaque sizes for each DENV type are highlighted in red boxes. **(B)** Median plaque size (µm^2^) characterized from images at increasing days post infection (DPI). 1E4 µm^2^ was established as the target plaque size and was indicated by the red dotted line. **(C)** DENV-1 viral adsorption and inoculum volume. Average PFU/well vs 1:2 serial dilution factor as a function of inoculum volume (µL), by adsorption time (1 hour, 2 hours, 4 hours). Dotted lines represent a range of 5-199 plaques per well that ensures accurate plaque counting. **(D)** Average median plaque size (µm^2^) and average viral titer (PFU/mL) according to increased cells seeded per plate well for all four DENV serotypes. Dotted lines indicate the optimized cell seeding parameter of 40,000 cells per well.

Infection incubation is the most time-consuming procedure in many plaque assays. It is also the critical step that allows plaques to grow and develop into optimal sizes for characterization. If plaque sizes are on the lower end of the scale, distinguishing plaque patterns from the background or artifacts is challenging. On the other hand, overly large plaques limit the total plaque number that can be precisely counted in each well, which translates to a tighter working concentration range and limited assay efficiency. Our goal was to limit the incubation time to improve throughput while maintaining high accuracy and sensitivity of the assay. Multiple assay parameters have been evaluated including infection time, viral adsorption time, inoculum volume and cell seeds per well. These parameters were optimized in sequence instead of tested in a DoE to limit the amount of consumed sample. Infection incubation time was optimized first as one of the decisive factors for this high-throughput assay’s run duration. As shown in **Figure 3A**, µPlaque images were captured after 2 to 5 days post infection for each DENV type. Inoculum volume was 25 µL and adsorption time was kept at 4 hours. It is shown that the viral spreading process progresses differently depending on the serotype. Plaques developed faster in DENV-2 and DENV-4 samples compared to DENV-1 and DENV-3. DENV-3 was the slowest, which was confirmed by both the fluorescent images and calculated median plaque sizes. The similar plaque growth rates between DENV-2 and DENV-4 are likely associated with their similar lineage. Dengue viral genome is an RNA that encodes non-structural proteins as a backbone and structural proteins including the capsid (C), the membrane (prM) and the envelope (E) proteins. DENV-2 tested in this study from TV003/TV005 is chimeric with DENV-4 where only the prM and E genes of DENV-4 were substituted by those of DENV-2 (37). Between DENV-2 and DENV-4 samples, small satellites of plaques appeared in DENV-2 images. 1E4 µm^2^ was the target plaque size from the automated counting algorithm (i.e. Kaleido 3.0, PerkinElmer) based on the principle described above, which takes 2 days for DENV-1, DENV-2 and DENV-4 while 3 days for DENV-3 to achieve (**Figure 3B**).

Next, viral adsorption time was optimized as a function of the dilution factor with DENV-1 samples. Time of incubation was kept at 2 days. Dilution linearity was observed as shown in **Figure 3C** from all three adsorption times including 1-, 2- and 4-hour under log_2_ scales for both axes. As expected, viral titer results trend up with increased adsorption time. We also noticed that higher errors appear around higher dilution factor points for 1- and 2-hour conditions. This can be explained by increased relative counting errors at the lower end of plaque numbers per well from the automated counting algorithm. Therefore, 4 hours was selected as the optimized adsorption incubation time. Under 4-hour adsorption time, potency results between 25 µL and 40 µL inoculum volumes are comparable and higher than the results from 18 µL inoculum. 25 µL condition was selected as it improves viral infection and prevents wells from drying compared to 18 µL, while saving sample volume compared to 40 µL. Additionally, as discussed in **Figure 2C**, 25 µL inoculum improves counting accuracy compared to 40 µL for DENV-2 sample, which reaffirms that 25 µL is the optimal inoculum among other tested volumes.

Cells seeded per plate well were also investigated to ensure a confluent cell monolayer for viral infection and plaque development. As shown in **Figure 3D**, the number of seeded cells varied from 20,000 to 90,000 per well. Inoculum volume and incubation time were kept at25 µL and 2 days, respectively. The averaged median plaque size remained at a similar level for DENV-1 while decreased for all three other DENV types as cell amount increased in individual wells. Averaged titers (PFU/mL) stayed stable in the 20,000 to 60,000 cells per well range for all DENV types and gradually declined as cell amount continued to increase above 60,000 per well. Seeding cell density was chosen as 40,000 to ensure a ~95% confluent cell layer, a robust potency report range (i.e. minimal result changes with cell density varying within ±50% from the target value) and suitable plaque sizes for the automated counting algorithm.

### 3.3 Assay robustness

**Figure 4:**
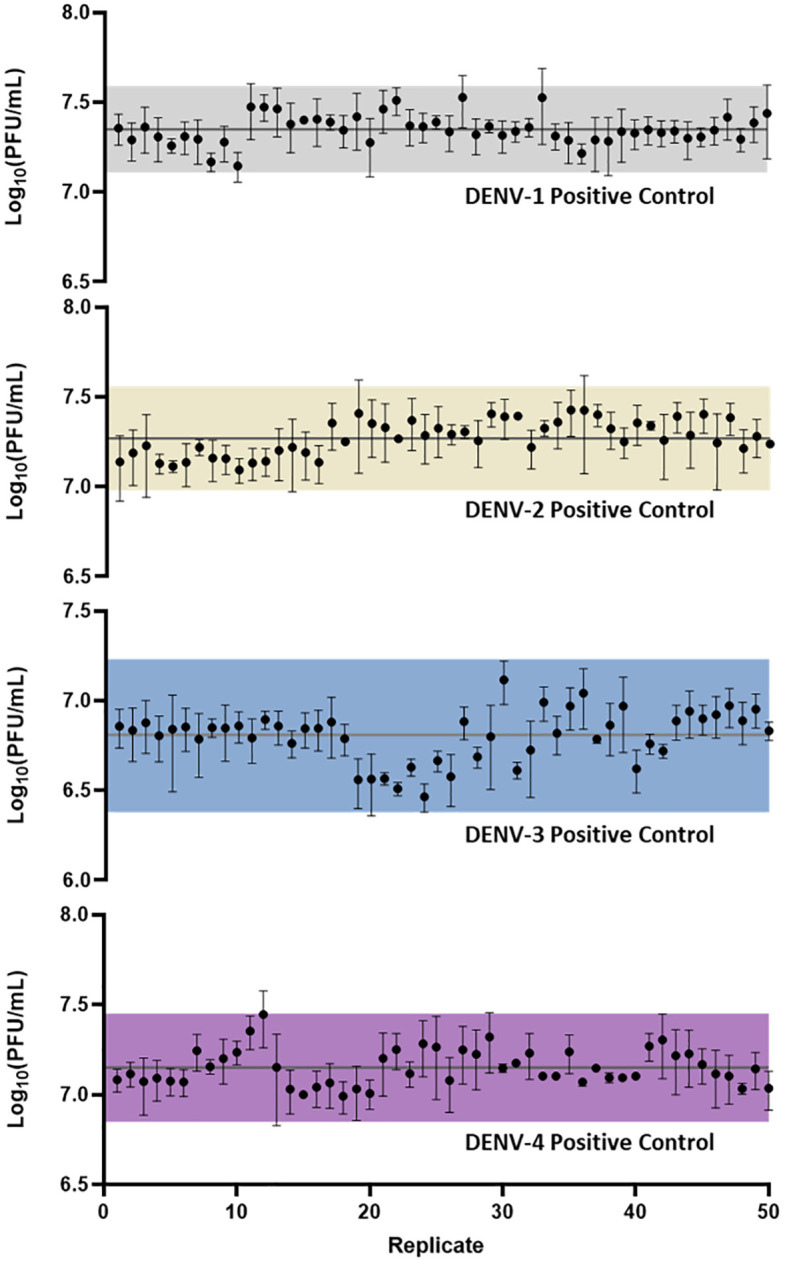
µPlaque assay repeatability and intermediate precision. 50 replicates of positive control samples for each serotype were tested in different runs by multiple analysts through a duration of 9 months. Error bar of each point represents the standard deviation from multiple serials diluted points of each individual sample. Solid horizontal lines in each subplot refer to the averaged viral titer results. Shaded regions represent a three-sigma range calculated from the dataset.

Following assay optimization, a full µPlaque assay automation protocol was established in the integrated robotic system as described in the Materials and Methods section. The overall assay duration is 4 or 5 days depending on the sample serotype. A full run includes 24 96-well microplates, with two positive controls (PCs) and 14 samples of the same serotype on each plate. Every PC and sample were pre-diluted and then serial diluted into a total of six wells. These six data points were back-calculated with dilution factors and averaged to report the final titer of a PC or a sample. The repeatability and intermediate precision of µPlaque assay were demonstrated with PC results in **Figure 4** for all four DENV serotypes. The exhibited dataset includes 50 PC replicates of each serotype, which were tested in 4-6 separate runs by 3-4 different analysts over a 9-month duration. The averaged relative standard deviations (RSD) of each serotype are 22.1%, 24.4%, 24.7%, and 22.5% from different diluted points of individual PC replicates, demonstrating a lower level of assay variability compared to manual methods (38). Averaged viral titers in log_10_ (PFU/mL) are 7.35, 7.27, 6.81, and 7.15 for DENV-1 to DENV-4, respectively. A three-sigma range was used to establish the assay acceptance range. They are [7.11, 7.59], [6.98, 7.56], [6.38, 7.23] and [6.85, 7.45] for DENV-1 to DENV-4 accordingly. No trending was observed within the dataset, indicating assay consistency from various plates, over multiple cell trains, and among different analysts on different experiment dates. Therefore, we confirmed that this automated assay has adequate repeatability and intermediate precision for dengue viral titer testing.

### 3.4 Concordance with 24-well manual assay

**Figure 5:**
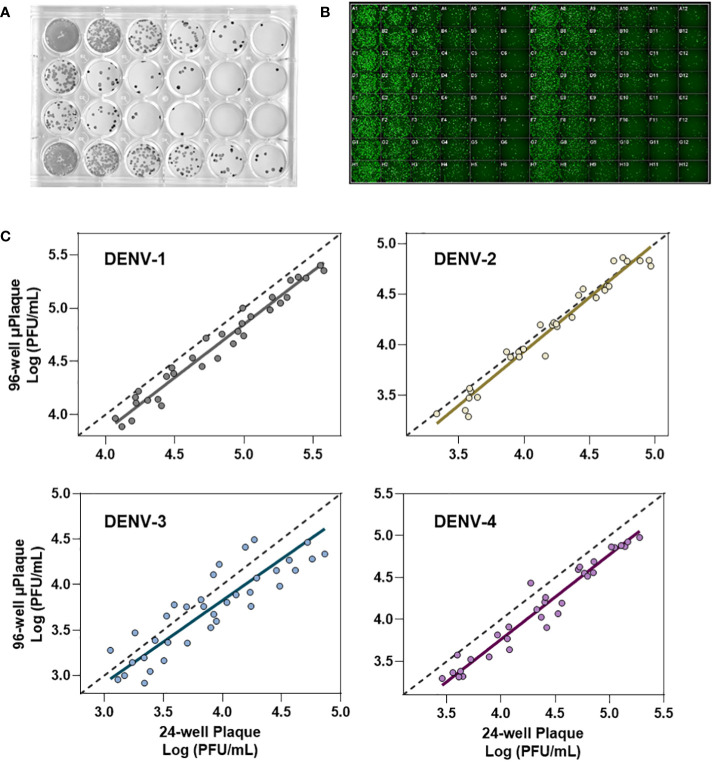
Concordance with 24-well manual assay. **(A)** Manual 24-well plaque assay. Plaques were visualized with DAB/HRP conjugate. Viral titration from left to right. **(B)** Automated 96-well plaque assay. Plaques stained via AlexaFluor488. Viral titration, left to right, starting in columns 1 and 7. **(C)** Concordance study between manual and automated plaque assays for each serotype.

We next evaluated the suitability of utilizing the µPlaque assay to test tetravalent drug product samples. The evaluation was carried out by measuring a set of samples via a traditional 24-well manual plaque assay, and comparing to the high throughput µPlaque assay. These two assays utilize the same serotype-specific primary antibodies but have intrinsic differences in assay parameters. Thus, we expected different viral titers from the same sample with a high correlation between two assays. Selected assay parameters are compared in **Table 2**, which are expected to influence plaque assay results from the same sample due to differences in surface area, inoculum volume, and timing of viral adsorption (36, 39). Example plate-view results for both assays are shown in **Figures 5A, B**. A clear trend of decreased plaque counts from the left to right of each plate can be observed that corresponds to increased dilution factors. We further carried out a concordance study with a sample set consisting of three formulations containing DENV-1 to DENV-4, with each formulation prepared at several different drug product concentrations in both liquid and lyophilized formats. The µPlaque assay showed a positive correlation that trended with the 24-well manual plaque method. As shown in **Figure 5C**, PFU/mL values from µPlaque assay are slightly lower than the results from 24-well manual plaque assay for DENV serotypes 1, 3 and 4, while the results are more aligned for DENV-2 samples. Some discrepancies were observed under the 2E3 PFU/mL, where the µPlaque assay did not have any valid results due to limited plaques developed. This suggests µPlaque method has lower sensitivity compared to the manual plaque, which can be attributed to the shorter viral incubation time, smaller surface area and plaque patterns within the µPlaque format. As a result, µPlaque assay range was established at above 2E3 PFU/mL for all serotypes.

**Table 2:** Assay parameter comparison.

Parameter	Manual (24-well)	µPlaque (96-well)	Scale Factor
Surface area per well (cm^2^)	1.90	0.32	6
Inoculum Volume (µL)	100	25	4
Avg. Depth of Inoculum (mm)	0.53	0.78	0.68
Cells per well	4E5	4E4	10
Cells/cm^2^	2.1E5	1.3E5	1.68
Adsorption Time (h)	1	4	0.25
Days Post Infection (DPI) to visualize plaques	6 days	2/3 days	N/A

The Deming regression between µPlaque and manual plaque results within the established range. The fitted slopes and their 95% confidence intervals (CIs) are 1.001 [0.9360, 1.066] for DENV-1; 1.068 [0.9721, 1.165] for DENV-2; 0.9092 [0.7326, 1.086] for DENV-3 and 1.012 [0.9509 to 1.072] for DENV-4. The slope of regression is not significantly different from 1.000 across all serotypes. These data suggest that there is no significant proportional bias, or in other words, the PFU/mL results from µPlaque change in the same scale as the titer values from 24-well manual assay change. In general, titer results from the 96-well µPlaque assay tend to be lower than the titers from the 24-well manual plaque assay. The offsets between two assays are 1.4-fold, 1-fold, 1.6-fold, and 1.9-fold for DENV-1 to DENV-4 respectively. These results demonstrated that µPlaque assay is a concordant method compared to the traditional 24-well manual plaque method.

### 3.5 Tetravalent investigation

**Figure 6:**
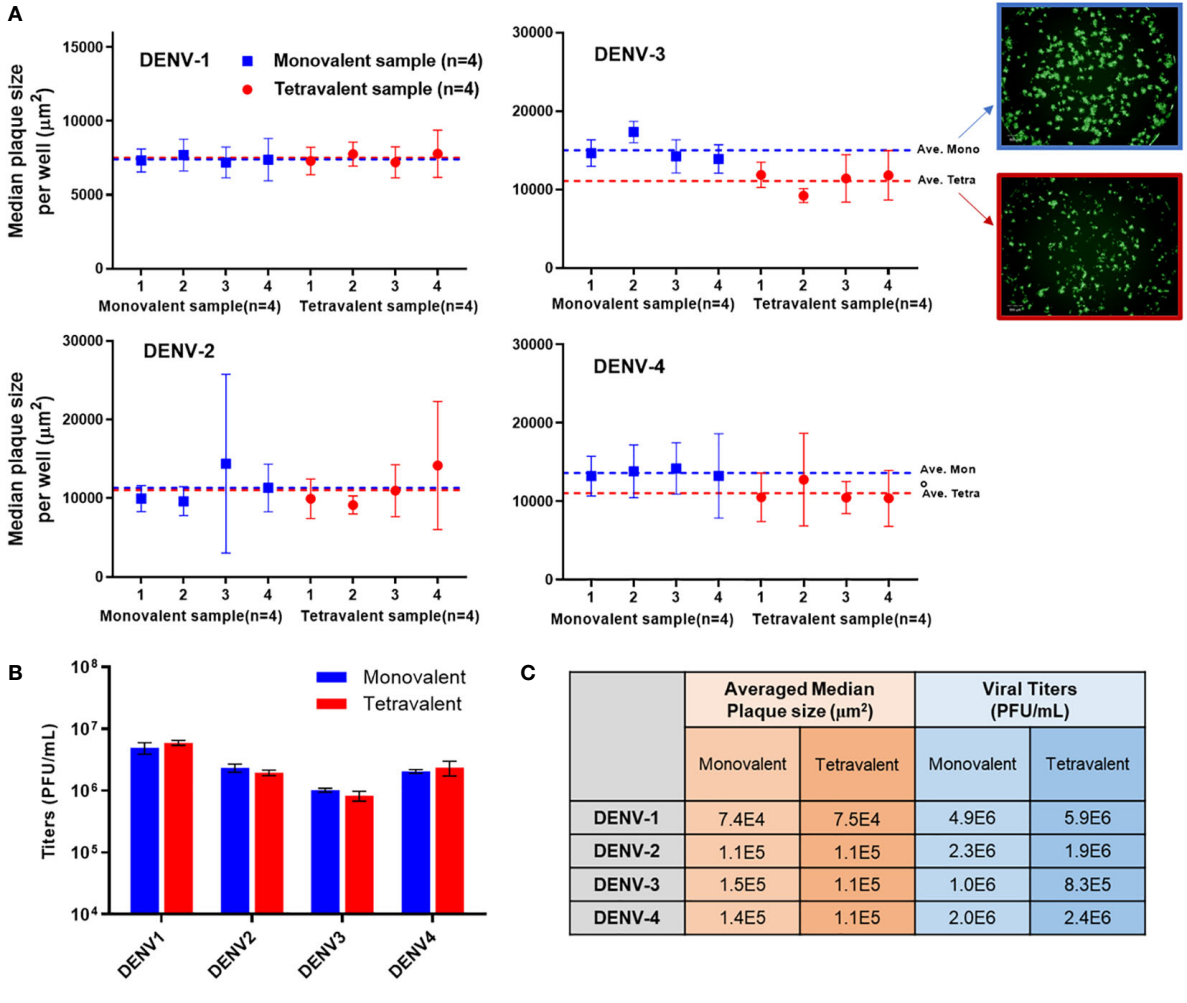
µPlaque assay capability in measuring tetravalent sample titers. **(A)** Averaged median plaque sizes per well for monovalent and tetravalent samples tested for each DENV type with example images for DENV-3. Dotted lines represent the averaged median plaque size per well for all monovalent (n=4) and all tetravalent (n=4) samples in blue and red respectively. **(B)** Viral titer (PUF/mL) results for four DENV serotypes from monovalent and tetravalent samples. **(C)** A comparison between the plaque size and viral titer results shown in subplot A and B for all serotypes. Statistical analysis was performed between monovalent and tetravalent samples results for each serotype in both averaged median plaque size and viral titer.

Analytical testing for tetravalent samples is required to support dengue vaccine development studies especially in the formulation area. When infected with tetravalent viral samples, Vero cells form plaques for all serotypes present instead of only the type being stained. These additional serotypes impact the growth of the type of interest often by crowding and limiting the plaque size. As DENV-3 grows relatively slower than the other types, it is especially susceptible to competition and growth suppression. Suppressed plaques will appear small and scattered when compared to ‘typical’ plaques. This adds difficulty in the automated plaque counting as more of those plaques might fall below the minimum size threshold, which leads to undercounting. To better understand the assay sensitivity, monovalent and tetravalent samples with the same target viral titer of each serotype were tested on the same µPlaque plate. The average median plaque size per well was calculated and plotted in **Figure 6A**. Plaque sizes were further averaged among all the monovalent (n=4) or tetravalent (n=4) samples of each serotype as indicated by dotted lines. Minimal plaque size differences were observed in DENV-1 and DENV-2 samples. For both DENV-3 and DENV-4, average plaque size is lower in the tetravalent samples which can be interpreted as plaque crowding. These differences are statistically significant with 95% confidence level from t-tests as described in section 2.4 for DENV-3 (P=0.0084) and DENV-4 (P=0.0147) respectively. As mentioned above, DENV-3 crowding is most likely due to the relatively slower plaque growth compared with other serotypes. The suppression observed in DENV-4 can be a result of its relatively large plaque size, which means it is harder for DENV-4 plaques to develop into large sizes with other serotypes’ plaque growing in the same well. Nevertheless, viral titer results are determined by plaque counts instead of plaque sizes. Potency (PFU/mL) results were then compared between monovalent and tetravalent samples for each serotype as illustrated in **Figure 6B**. The same type of t-test was performed and showed no significant difference in viral titer results between monovalent and tetravalent results, indicating the suitability of the µPlaque assay in testing tetravalent dengue vaccine samples (**Figure 6C**). Assay capability demonstrated here in accurately measuring the same serotype in both drug substance (monovalent) and drug product (multivalent) broadens the area of supported studies throughout vaccine produce development cycles.

### 3.6 Assay application

**Figure 7:**
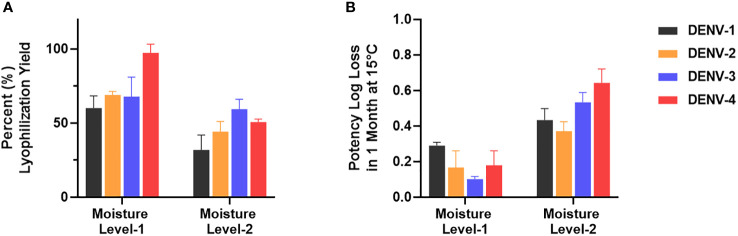
µPlaque assay application. **(A)** Lyophilization yield in potency compared to liquid formulation sample controls for all serotypes at two different moisture level (n=3). **(B)** Potency log loss of the same formulation samples held at moisture level-1 and level-2 after 1 month at 15°C. Each time point or condition has three samples tested (n=3). Error bars represent 2 times standard error from the mean.

µPlaque assay was applied to select a formulation condition from two moisture levels. In this study, we targeted two different moisture concentrations (i.e. level-1 and level-2) and examined their impact on potency yield post lyophilization (lyo) compared to liquid controls, as well as potency loss over 1 month at 15°C. The data shown in **Figure 7** illustrated significant benefit of moisture level-1 on both lyo yield and 1-month 15°C stability. This dataset demonstrates the need of a high-throughput assay for multivalent vaccine samples in formulation studies. Each sample not only needs to be tested for multiple conditions such as moisture level, temperate and storage time. The tetravalency of the dengue vaccine also further increases sample amount. In addition to formulation sample testing, µPlaque assay has also supported various process studies from upstream fermentation to downstream purification with monovalent dengue vaccine samples. More recently, µPlaque was adapted to support potency measurement for process development of a SARS-CoV-2 vaccine candidate. µPlaque assay allowed for high-throughput titer analysis in this work and accelerated the development and optimization of an industrial scale closed fully disposable microcarrier manufacturing process for LVV (28).

Potency measurement remains at the center of quality and efficacy characterizations for many pharmaceutical products especially vaccines (40). The need of rapid vaccine development has appeared increasingly with the COVID-19 pandemic as an example. The shorter product development cycle naturally raises expectations in expedited traditional analytical methods such as the manual plaque assay for viral titer test. More specifically, traditional experimental design space for LVV products within vaccine bioprocess can be exceptionally large and heavily multi-dimensional within a single functional space (upstream, downstream, and drug product development). There can also be codependence and parallel activities performed across functional areas, which are usually time-sensitive and form a highly complex design space that requires understanding and optimization prior to project progression. The ability to quickly probe the knowledge space of LVV projects necessitates the use of high-throughput analytics that supports high sample counts and rapid turnaround, especially for multivalent LVVs. Efforts towards developing faster, simpler, and higher throughput plaque assays have been reported to characterize viral samples such as Malaria (41), SARS-CoV-2 (25, 27), and RSV (26). Analytical challenges appeared from the tetravalent nature of the dengue vaccine. Development of a fully automated immuno-staining based µPlaque assay described in this work demonstrated multiple novel approaches that improve plaque assay throughput for dengue vaccine samples without sacrificing result accuracy. First, the incubation time of this assay was shortened from 6 days of a traditional 24-well manual plaque assay to 2-3 days depending on serotype. Secondly, 96-well microplates allow for more sample slots as well as the capability of including positive controls on each plate. Additionally, a fully automated robotic system together with an automated plaque counting algorithm requires minimal analysts’ supervision and ensures highly repeatable and precise potency measurement.

Ongoing efforts to further improve the µPlaque assay include neutralizing antibody application to limit serotype competition, multiplex assay development, and a deep learning-based plaque counting algorithm. To start with, competition was observed in tetravalent sample testing with the current assay format leading to relatively slower plaque growth in DENV-3 and DENV-4 as discussed in section 3.5. These concomitant effects can be limited by adding serotype-specific neutralizing antibodies in the diluent which neutralizes complementary types from the serotype of interest. Secondly, the multiplexing development will enable multiple stainings for the same multivalent sample. More specifically, a tetravalent dengue vaccine sample that contains all four serotypes can be stained with four different type-specific mAbs, followed by secondary antibody staining and imaging at four different wavelengths. Four major fluorescent channels including DAPI, GFP, yellow fluorescent protein (YFP) and Cy5 will be utilized for the four serotypes to limit overlapped fluorescent signals. In order to reduce cross-reactivity, applicable primary mAbs can either be directly labeled or from different origins, or different isotypes within the same species. Developing a multiplexing µPlaque method as described will further improve the assay throughput and minimize consumed sample volume by four times for formulation samples, which translates to 1536 samples instead of 384 samples tested in each run. Lastly, we have also been developing a custom automated image analysis method to improve the robustness of plaque counting, and to release the burden of manual image examination during troubleshooting. A novel deep learning-based pipeline will further improve the plaque counting method from the commercial software currently employed by µPlaque assay. The algorithm has shown promising results in accurately differentiating plaques from artifacts in cell plate wells, and the ability of separating partially fused plaque patterns. Further optimization will allow a cell layer confluency check and seamless implementation into the current assay workflow. It is also our aim to generalize the algorithm for other cell-based assay image analysis.

## 4 Conclusions

A fully automated 96-well microplate potency method termed the µPlaque assay was developed for viral titer measurements to support the development of a live-attenuated tetravalent dengue vaccine. Assay optimization was carried out for various parameters including viral adsorption time and infection time, cell seeding density, and inoculum volume. Assay robustness was demonstrated by monitoring positive control results tested by multiple analysts from different runs over a 9-month duration. A concordance study to compare µPlaque assay with a traditional manual plaque assay was performed and proved the potency result concordance between the two methods. Full automation of the µPlaque assay via an integrated robotic system and automated plaque counting method shortened the run time by half and improved sample throughput by more than ten times compared to the manual plaque method, which illustrated the potential and impact of high-throughput cell-based analytics in vaccine development space. Moreover, µPlaque assay exhibited the capability of reporting accurate potency for individual serotypes from tetravalent dengue vaccine samples. Ongoing efforts including multiplexing assay development and deep learning-based plaque counting algorithm are to further improve the assay accuracy and efficiency. This work showcased, for the first time in the literature, the development and application of a fully automated cell-based assay enabled by an integrated robotic system, and its potential in advancing the high-throughput analytics field which leads to accelerated vaccine development.

## Data availability statement

The original contributions presented in the study are included in the article/supplementary material. Further inquiries can be directed to the corresponding authors.

## Ethics statement

Ethical approval was not required for the studies on animals in accordance with the local legislation and institutional requirements because only commercially available established cell lines were used.

## Author contributions

YW: Data curation, Visualization, Writing – original draft, Writing – review & editing. MT: Conceptualization, Data curation, Investigation, Methodology, Visualization, Writing – review & editing. CH: Data curation, Investigation, Methodology, Writing – review & editing. AG: Data curation, Investigation, Writing – original draft. LS: Formal analysis, Writing – review & editing. RL: Data curation, Investigation, Writing – review & editing. HP: Supervision, Writing – review & editing. KK: Methodology, Supervision, Writing – review & editing. PD: Conceptualization, Project administration, Writing – review & editing. JL: Supervision, Writing – review & editing.

## References

[1] Simmons CP Farrar JJ Nguyen v V Wills B. Dengue. N Engl J Med (2012) 366(15):1423–32. doi: 10.1056/NEJMra1110265 22494122

[2] Wilder-Smith A Ooi EE Horstick O Wills B. Dengue. Lancet (2019) 393(10169):350–63. doi: 10.1016/S0140-6736(18)32560-1 30696575

[3] Brady OJ Gething PW Bhatt S Messina JP Brownstein JS Hoen AG. Refining the global spatial limits of dengue virus transmission by evidence-based consensus. PloS Negl Trop Dis (2012) 6(8):e1760. doi: 10.1371/journal.pntd.0001760 22880140 PMC3413714

[4] Dengue and Severe Dengue . World Health Organization. Available at: https://www.who.int/news-room/fact-sheets/detail/dengue-and-severe-dengue (Accessed May 14, 2023).

[5] Deng SQ Yang X Wei Y Chen JT Wang XJ Peng HJ. "A review on dengue vaccine development. " Vaccines (Basel) (2020) 8(1):63. doi: 10.3390/vaccines8010063 PMC7159032 32024238

[6] Pintado Silva J Fernandez-Sesma A. Challenges on the development of a dengue vaccine: A comprehensive review of the state of the art. J Gen Virol (2023) 104(3):001831 doi: 10.1099/jgv.0.001831 36857199 PMC10228381

[7] Thomas SJ. The necessity and quandaries of dengue vaccine development. J Infect Dis (2011) 203(3):299–303. doi: 10.1093/infdis/jiq060 21208919 PMC3071120

[8] Coller BA Clements DE Bett AJ Sagar SL Ter Meulen JH. The Development of Recombinant subunit envelope-based vaccines to protect against dengue virus induced disease. Vaccine (2011) 29(42):7267–75. doi: 10.1016/j.vaccine.2011.07.021 PMC3179979 21777637

[9] Manoff SB Sausser M Falk Russell A Martin J Radley D Hyatt D. Immunogenicity and safety of an investigational tetravalent recombinant subunit vaccine for dengue: Results of a phase I randomized clinical trial in flavivirus-naive adults. Hum Vaccin Immunother (2019) 15(9):2195–204. doi: 10.1080/21645515.2018.1546523 PMC6773383 30427741

[10] Russell KL Rupp RE Morales-Ramirez JO Diaz-Perez C Andrews CP Lee AW. A Phase I Randomized, Double-Blind, Place-bo-Controlled study to evaluate the safety, tolerability, and immunogenicity of a Live-Attenuated quadrivalent dengue vaccine in Flavivirus-Naive and Flavivirus-Experienced healthy adults. Hum Vaccin Immunother (2022) 18(5):2046960. doi: 10.1080/21645515.2022.2046960 35290152 PMC9225326

[11] McFarland R Verthelyi D Casey W Arciniega J Isbrucker R Schmitt M. Non-Animal replacement methods for human vaccine potency testing: State of the science and future directions. Proc Vaccinol (2011) 5:16–32. doi: 10.1016/j.provac.2011.10.002 PMC7129268 32288913

[12] Shank-Retzlaff M Wang F Morley T Anderson C Hamm M Brown M. Correlation between mouse potency and *in vitro* relative potency for human papillomavirus type 16 Virus-Like particles and Gardasil (R) vaccine samples. Hum Vaccines (2005) 1(5):191–97. doi: 10.4161/hv.1.5.2126 17012876

[13] McVey DS Galvin JE Olson SC. A Review of the effectiveness of vaccine potency control testing. Int J Parasitol (2003) 33(5-6):507–16. doi: 10.1016/S0020-7519(03)00067-5 12782051

[14] Verch T Trausch JJ Shank-Retzlaff M. Principles of vaccine potency assays. Bioanalysis (2018) 10(3):163–80. doi: 10.4155/bio-2017-0176 29333863

[15] Moe JB Lambert RD Lupton HW. Plaque assay for ebola virus. J Clin Microbiol (1981) 13(4):791–3. doi: 10.1128/jcm.13.4.791-793.1981 PMC273881 7014628

[16] Husson-van Vliet J Colinet G Yane F Lemoine P. A Simplified plaque assay for varicella vaccine. J Virol Methods (1987) 18(2-3):113–20. doi: 10.1016/0166-0934(87)90116-9 2828397

[17] Mann GF Allison LM Copeland JA Agostini CF Zuckerman AJ. A simplified plaque assay system for measles virus. J Biol Stand (1980) 8(3):219–25. doi: 10.1016/S0092-1157(80)80037-0 7410445

[18] Shurtleff AC Biggins JE Keeney AE Zumbrun EE Bloomfield HA Kuehne A. Standardization of the filovirus plaque assay for use in preclinical studies. Viruses (2012) 4(12):3511–30. doi: 10.3390/v4123511 PMC3528277 23223188

[19] Cooper PD. The plaque assay of animal viruses. Adv Virus Res (1961) 8:319–78. doi: 10.1016/S0065-3527(08)60689-2 13881155

[20] Elahi SM Nazemi-Moghaddam N Gadoury C Lippens J Radinovic S Venne MH. A rapid Focus-Forming Assay for quantification of infectious adenoviral vectors. J Virological Methods (2021) 297:114267. doi: 10.1016/j.jviromet.2021.114267 34437873

[21] Feitosa-Suntheimer F Zhu Z Mameli E Dayama G Gold AS Broos-Caldwell A. Dengue Virus-2 Infection affects fecundity and elicits specific transcriptional changes in the ovaries of aedes aEgypti mosquitoes. Front Microbiol (2022) 13:886787. doi: 10.3389/fmicb.2022.886787 35814655 PMC9260120

[22] Baer A Kehn-Hall K. Viral concentration determination through plaque assays: Using traditional and novel overlay systems. J Vis Exp (2014) 93:e52065. doi: 10.3791/52065 PMC4255882 25407402

[23] LaBarre DD Lowy RJ. Improvements in methods for calculating virus titer estimates from tcid50 and plaque assays. J Virol Methods (2001) 96(2):107–26. doi: 10.1016/S0166-0934(01)00316-0 11445142

[24] Sullivan K Kloess J Qian C Bell D Hay A Lin YP. High Throughput virus plaque quantitation using a flatbed scanner. J Virol Methods (2012) 179(1):81–9. doi: 10.1016/j.jviromet.2011.10.003 22044905

[25] Muruato AE Fontes-Garfias CR Ren P Garcia-Blanco MA Menachery VD Xie X. A High-Throughput neutralizing antibody assay for Covid-19 diagnosis and vaccine evaluation. Nat Commun (2020) 11(1):4059. doi: 10.1038/s41467-020-17892-0 32792628 PMC7426916

[26] Wen Z Citron M Bett AJ Espeseth AS Vora KA Zhang L. Development and application of a higher throughput rsv plaque assay by immunofluorescent imaging. J Virol Methods (2019) 263:88–95. doi: 10.1016/j.jviromet.2018.10.022 30381239

[27] Amarilla AA Modhiran N Setoh YX Peng NYG Sng JDJ Liang B. An optimized high-throughput immuno-plaque assay for Sars-Cov-2. Front Microbiol (2021) 12:625136. doi: 10.3389/fmicb.2021.625136 33643253 PMC7906992

[28] Ton C Stabile V Carey E Maraikar A Whitmer T Marrone S. Development and scale-up of Rvsv-Sars-Cov-2 vaccine process using single use bioreactor. Biotechnol Rep (Amst) (2023) 37:e00782. doi: 10.1016/j.btre.2023.e00782 36687766 PMC9841742

[29] Patel A Erb SM Strange L Shukla RS Kumru OS Smith L. Combined Semi-Empirical screening and design of experiments (Doe) approach to identify candidate formulations of a Lyophilized Live attenuated tetravalent viral vaccine candidate. Vaccine (2018) 36(22):3169-79. doi: 10.1016/j.vaccine.2017.04.086 28506515

[30] Lippi G Da Rin G. Advantages and limitations of total laboratory automation: a personal overview. Clin Chem Lab Med (CCLM) (2019) 57(6):802–11. doi: 10.1515/cclm-2018-1323 30710480

[31] Holland I Davies JA. Automation in the life science research laboratory. Front Bioengineering Biotechnol (2020) 8:571777. doi: 10.3389/fbioe.2020.571777 PMC7691657 33282848

[32] Daniels C Rodriguez J Lim E Wenger M. An integrated robotic system for high-throughput process development of cell and virus culture conditions: Application to biosafety level 2 live virus vaccines. Engineering in Life Sciences (2016) 16(2):202–9. doi: 10.1002/elsc.201400245

[33] Hansel CS Plant DL Holdgate GA Collier MJ Plant H. Advancing automation in high-throughput screening: Modular unguarded systems enable adaptable drug discovery. Drug Discovery Today (2022) 27(8):2051–6. doi: 10.1016/j.drudis.2022.03.010 35304338

[34] Roehrig JT Hombach J Barrett AD. Guidelines for plaque-reduction neutralization testing of human antibodies to dengue viruses. Viral Immunol (2008) 21(2):123–32. doi: 10.1089/vim.2008.0007 18476771

[35] Clements DE Coller BAG Lieberman MM Ogata S Wang G Harada KE. Development of a recombinant tetravalent dengue virus vaccine: immunogenicity and efficacy studies in mice and monkeys. Vaccine (2010) 28(15):2705–15. doi: 10.1016/j.vaccine.2010.01.022 PMC2837772 20097152

[36] Mittereder N March KL Trapnell BC. Evaluation of the concentration and bioactivity of adenovirus vectors for gene therapy. J Virol (1996) 70(11):7498–509. doi: 10.1128/jvi.70.11.7498-7509.1996 PMC190817 8892868

[37] Hou J Ye W Chen J. Current development and challenges of tetravalent live-attenuated dengue vaccines. Front Immunol (2022) 13:840104. doi: 10.3389/fimmu.2022.840104 35281026 PMC8907379

[38] Payne AF Binduga-Gajewska I Kauffman EB Kramer LD. Quantitation of flaviviruses by fluorescent focus assay. J virological Methods (2006) 134(1-2):183–9. doi: 10.1016/j.jviromet.2006.01.003 16510196

[39] Allison AC Valentine RC. Virus particle adsorption: III. Adsorption of viruses by cell monolayers and effects of some variables on adsorption. Biochim Biophys Acta (1960) 40:400–10. doi: 10.1016/0006-3002(60)91380-9 13792819

[40] Sanyal G. Development of functionally relevant potency assays for monovalent and multivalent vaccines delivered by evolving technologies. NPJ Vaccines (2022) 7(1):50. doi: 10.1038/s41541-022-00470-4 35513416 PMC9072649

[41] Thomas JA Collins CR Das S Hackett F Graindorge A Bell D. Development and application of a simple plaque assay for the human malaria parasite plasmodium falciparum. PloS One (2016) 11(6):e0157873. doi: 10.1371/journal.pone.0157873 27332706 PMC4917082

